# Elevated Thyroglobulin Antibody Level is Associated with Decreased Anti-Müllerian Hormone in Women of Reproductive Age

**DOI:** 10.1155/2023/1861752

**Published:** 2023-12-13

**Authors:** Jazyra Zynat, Xinling Wang, Li Han, Shuqing Xing, Guzailinuer Jvlaiti, Qingqing Liu, Lingling Dong, Yanying Guo

**Affiliations:** Department of Endocrinology, People's Hospital of Xinjiang Uygur Autonomous Region, China

## Abstract

**Purpose:**

Women with Hashimoto's thyroiditis (HT) have an increased risk of ovarian insufficiency. However, whether thyroid antibodies affect the ovarian reserve remains controversial. The aim of this study was to explore the possible relationship between anti-Müllerian hormone (AMH) and thyroid peroxidase antibody (TPOAb) and thyroglobulin antibody (TgAb) levels in women of reproductive age.

**Methods:**

A total of 483 women between 18 and 45 years old who had their TPOAb, TgAb, thyroid-stimulating hormone (TSH), free thyroxine (FT4), and AMH levels measured on the same day were enrolled in this study. The levels of TSH, FT4, TPOAb, and TgAb, the prevalence of overt and subclinical hypothyroidism, and the positive rate of TPOAb and TgAb were compared between patients with low (below the 10th percentile), normal (10th to 90th percentile), and high (higher than the 90th percentile) AMH levels.

**Results:**

The median AMH level was 1.72 (0.33–4.27) ng/mL. A total of 9.9% of patients had low AMH levels. The TgAb levels and the prevalence of TgAb positivity were higher in the low AMH group (37.62 (13.10–232.68) IU/mL, 35.42%) than in the normal (12.46 (10.0–67.04) IU/mL, 19.59%) and high (13.61 (10.0–95.74) IU/mL, 23.4%) AMH groups (*p*=0.001, *p*=0.040, respectively). Serum AMH levels were inversely correlated with TgAb levels (*r* = −0.114, *p*=0.013).

**Conclusion:**

The AMH of women of reproductive age is affected by HT. Furthermore, women with the lowest AMH level had higher levels of TgAb and a positive rate of TgAb, and high TgAb levels may cause autoimmune damage to the ovaries.

## 1. Introduction

Autoimmune thyroid disease (AITD) is the most common autoimmune disease among women of reproductive age, affecting 5%–20% of the female population [1]. A comprehensive review of multiple studies on autoimmune thyroid disease in infertile women showed that the overall relative risk of infertility is 2.1% compared to that of control patients [2]. One of the most common AITDs is Hashimoto thyroiditis (HT), which is characterized by the presence of thyroid peroxidase antibody (TPOAb) and thyroglobulin antibody (TgAb) [3]. It is well known that menstrual cycle disturbances in patients with HT will increase by as much as 3 times [4]. Patients with autoimmune thyroid disease have ovarian dysfunction and an increased frequency of miscarriage and infertility [5]. Even in women with HT who have normal thyroid function, the presence of thyroid autoantibodies is also associated with female infertility [6].

The ovarian reserve is essential for reproductive function, reflecting the quantity and quality of oocytes in the follicular pool [7], and women with decreased ovarian follicles have an increased risk of premature ovarian failure (POF) [8]. There are many markers for evaluating ovarian reserve, such as ovarian volume, antral follicle count (AFC), estradiol (E2), and serum follicle-stimulating hormone (FSH) concentrations [9], as well as inhibin B and anti-Müllerian hormone (AMH) concentrations *n* [10]. AMH is produced by the granulosa cells of primary, preantral, and small antral follicles in the ovaries *n* [11] and could reflect the total number of antral and preantral follicles, thereby serving as a marker of ovarian reserve [11, 12]. Compared with other biological indicators, AMH has obvious advantages in the assessment of ovarian reserve and is the most accurate biomarker for ovarian senescence [13]. In addition, AMH levels in blood are not affected by the menstrual cycle, remaining stable throughout the cycle [14]. Multicenter clinical studies MERIT and MEGASET showed that using AMH as an indicator is better in almost every research center than AFC, and the consistency of AMH is better [15]. The MERIT and MEGASET studies found that, with known AFC, adding AMH detection will increase the predictive value, but the addition of AFC information in known AMH does not increase the predictive value [16]. Compared with FSH, the AMH index is more sensitive and has higher specificity, which can help clinical patients to find POF patients earlier [16].

Studies have shown that abnormal thyroid function directly affects the reproductive system via thyroid hormone receptors on the surface of oocytes [17]. Moreover, studies have shown that thyroid autoimmune diseases may be related to systemic autoimmunity, which leads to POF [18, 19]. There are many studies related to hypothyroidism and ovarian function or thyroid-stimulating hormone and AMH. However, whether and how thyroid autoimmunity affects ovarian reserve remains controversial. Moreover, TPOAb and TgAb are both markers of thyroid autoimmunity. As far as we know, there are few and inconsistent reports on the interaction between TgAb and AMH. Some studies reported a negative correlation between TgAb and AMH [20], while another study reported a positive correlation between them [21]. However, some studies have reported that there is no correlation between TgAb and AMH [4, 6]. But it is worth noting that whether there is a correlation between them and what kind of relationship they have, and the underlying mechanism is not clear. Taking into account the above evidence, we conducted a retrospective cross-sectional analysis to investigate the relationship of HT, as assessed by thyroid antibody (TPOAb and TgAb) levels, and ovarian reserve, as measured by AMH concentration, among a general population of reproductive age women.

## 2. Methods

### 2.1. Study Population

The retrospective cross-sectional study was conducted with the women from two communities of Urumqi in Xinjiang Province (*n* = 461) and the women who visited People's Hospital of Xinjiang Uygur Autonomous Region between January 2016 and April 2019 (*n* = 485). Women were included in this study who were between 18 and 45 years old, had their AMH values measured with the same AMH assay, and had their AMH, TSH, FT4, TgAb, and TPOAb levels tested on the same day at People's Hospital of Xinjiang Uygur Autonomous Region. Due to the decrease in AMH levels when taking oral contraceptives or during pregnancy [22], women who used OC or were pregnant up to three months before testing were excluded. At the same time, we excluded patients with iatrogenic causes that could cause low ovarian reserve (ovarian endometriosis or removal of other benign ovarian surgery, oophorectomy, or gonadal toxicity treatment, i.e., chemotherapy or radiation therapy) and patients with known low ovarian reserve due to genetic causes (Turner syndrome, FMR1 premutation, abnormal karyotype, or any unbalanced translocation). Furthermore, women with a history of thyroid disease who were taking medication for hyperthyroidism or hypothyroidism and newly diagnosed thyroid disease who need medical treatment and self-reported other autoimmune diseases were excluded. A total of 490 women met the above criteria. Among them, 7 cases were excluded because of missing data. Finally, a total of 483 women were included in analysis of this study. The study was approved by the Ethics Committee of People's Hospital of Xinjiang Uygur Autonomous Region, and the procedures used in this study adhere to the tenets of the Declaration of Helsinki. All subjects provided written informed consent before participating in the study.

### 2.2. Measurements

Serum samples were drawn after patients fasted for at least 8 hours overnight, and then, the samples were stored at −20 °C; laboratory testing was completed within 2–4 hours. Serum FT4, TSH, TgAb, and TPOAb concentrations were measured by using a Roche electrochemiluminometric analyzer (Cobas-e601 analyzer, Roche, Mannheim, Germany). AMH levels were also measured by using an electrochemiluminometric analyzer (Cobas-e601 analyzer, Roche, Mannheim, Germany). AMH assays demonstrated stable intra-assay and interassay coefficients of variation of 4.9 and 7.0%, respectively.

### 2.3. Definitions

Hashimoto's thyroiditis was defined as high levels of TPOAb (>35 IU/mL) and/or TgAb (>116 IU/mL) (Roche).

Overt hypothyroidism was defined as thyrotropin >4.2 mIU/l and free *T*4 <11.5 pmol/l [23].

Subclinical hypothyroidism was defined as thyrotropin >4.2 mIU/l and free T4 within the normal range [23].

Low ovarian reserve: there is currently no consensus or uniformly accepted definition of low ovarian reserve in terms of AMH. However, in clinical practice, women who still have regular periods diagnose abnormal ovarian reserve by using various methods, such as elevated but not menopausal basal FSH levels, low AMH, low AFC, or a failed clomiphene citrate challenge test [24, 25]. For AMH, the Bologna criteria adopted a range between 0.5 and 1.1 ng/mL as the feature of an abnormal ovarian reserve test [23]. The reference interval of AMH specific to Chinese population derived from a large set of data has not been reported. Similarly, the reference range of AMH in Xinjiang has not been established yet. Thus, the whole study subjects in this study were divided into three different categories: (a) low AMH group (women with AMH below the 10th percentile of the values), (b) normal AMH group (women with AMH values from 10th to 90th percentile of the values), and (c) high AMH group (women with AMH values higher than the 90th percentile of the values). With respect to thyroid function, in the different AMH groups, subjects who are taking medication for hyperthyroidism or hypothyroidism are excluded from the calculation of median TSH and FT4. Similarly, as far as AMH is concerned, subjects with factors that may affect AMH levels are excluded from this study. The TPOAb and TgAb levels and the positive rate, along with the levels of TSH and FT4, were compared between women with different AMH level categories.

### 2.4. Statistical Analysis

Data analysis was performed with IBM SPSS Statistics, Version 21.0 (SPSS, Chicago, IL, USA). Descriptive variables with a normal distribution are presented as the mean with standard deviation (SD), and categorical variables with a nonnormal distribution are presented as the median (25th and 75th percentiles). Student's *t*-tests were used for comparison of continuous variables. Mann–Whitney *U* tests were used for nonnormally distributed continuous variables. ANOVA was used to test the significance of the mean difference of normally distributed variables between more than two groups. Kruskal–Wallis tests were used to compare the baseline values of skewed distribution variables. If there was a significant difference, the skewed distribution variable was ranked, ANOVA was conducted, and further pairwise comparisons were performed. Nominal variables were presented as the number of cases and their percentage (%). Significant differences between groups were analyzed using a chi-square test. Spearman's correlation analysis was used to analyze the correlation between AMH and TPO/TgAb levels. A *p* value <0.05 was considered statistically significant.

## 3. Results

**Figure 1:**
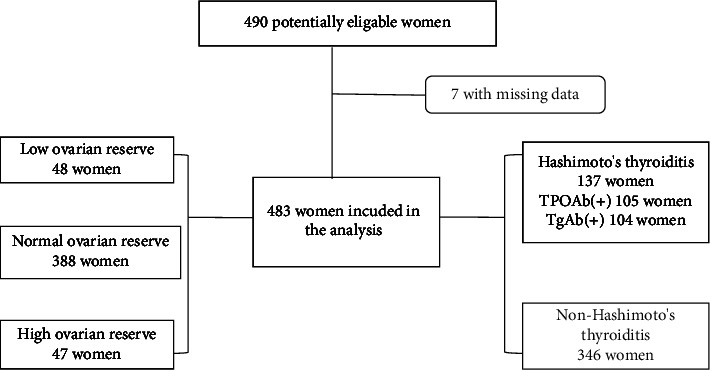
Flowchart of the study.

A total of 483 women, attending People's Hospital of Xinjiang Uygur Autonomous Region between January 2016 and April 2019, were eligible for the analysis of this study.  Figure 1 shows the flowchart of the study.

The mean age of the study participants was 33.77 ± 7.47 years. The median (interquartile range (IQR)) AMH level was 1.72 (0.33–4.27) ng/mL, the TSH level was 2.69 (1.80–4.24) *µ*IU/mL, TPOAb was 13.48 (9.02–26.38) IU/ml, and TgAb was 13.56 (10.00–82.06) IU/ml.  Table 1 shows the characteristics of all eligible women. There were 137 participants (28.4%) with HT. Among them, 105 participants were positive for TPOAb (21.7%) and 104 participants were positive for TgAb (21.5%) (Figure 1). The AMH levels of the low AMH group, normal AMH group, and high AMH group were 0.01 (0.01–0.02) ng/mL, 1.73 (0.53–3.83) ng/mL, and 9.08 (7.91–11.71) ng/mL, respectively, *p* < 0.001 (Table 1).

### 3.1. Thyroid Function and AMH

With respect to thyroid function, the level of FT4 in the low AMH group, the normal AMH group, and the high AMH group was 1.22 ± 0.29 ng/dl, 1.25 ± 0.44 ng/dl, and 1.33 ± 0.63 ng/dl, respectively. The FT4 levels were comparable between the low AMH group, normal AMH group, and high AMH group, *p*=0.482. Similarly, there was no significant difference in TSH levels between different AMH groups: low AMH group (3.02 (1.46–4.32)) *µ*IU/mL, normal AMH group (2.69 (1.81–4.21)) *µ*IU/mL, and high AMH group (2.31(1.70–4.18)) *µ*IU/mL, *p*=0.619 (Table 1). Furthermore, there was no statistically significant difference in the prevalence of overt and subclinical hypothyroidism between different AMH groups (overt hypothyroidism: 4.17% in low, 2.32% in normal, and 2.13% in high AMH groups, *p*=0.764; subclinical hypothyroidism: 31.25%, 25%, and 23.4% in low, normal, and high AMH groups, *p*=0.608) (Table 2).

### 3.2. Thyroid Autoimmunity and AMH

**Figure 2:**
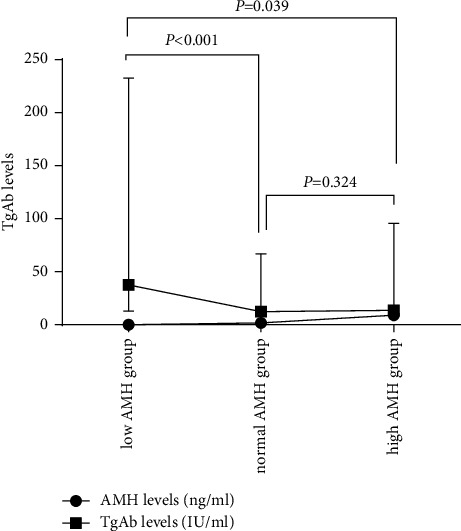
TgAb levels at different AMH level groups.

Regarding the thyroid autoimmunity, the level of TPOAb was 14.96 (8.66–119.93) IU/ml in low, 13.33 (8.87–24.93) IU/ml in normal, and 15.06 (10.24–25.72) IU/ml in high AMH groups; no statistically significant difference was found between different AMH groups, *p*=0.394. However, women in the low AMH group had a higher level of TgAb, 37.62 (13.1–232.68) IU/ml, than those in the normal and higher AMH groups, 12.46 (10–67.04) IU/ml and 13.61 (10–95.74) IU/ml, respectively (*p*=0.039, *p* < 0.001, Figure 2). Most importantly, the serum AMH levels were negatively correlated with TgAb levels (*r* = −0.114, *p*=0.013) but not with TPOAb levels (*r* = −0.024, *p*=0.599) (Table 3).

Plot graph of TgAb level and AMH level in low, normal, and high AMH groups. The *p* values in this figure are for TgAb levels. In different AMH level groups, the TgAb level of the low AMH group was the highest, which was statistically different from the TgAb level of the normal AMH group (*P* < 0.001) and the high AMH group (*P* = 0.039, respectively). When comparing TgAb levels between the normal and high AMH groups, no significant difference was found (*P* = 0.324).

The levels of AMH were significantly different in the patients with or without HT (*p*=0.007). Furthermore, we also compared the prevalence of HT, TPOAb-positive rate, and TgAb-positive rate in different AMH groups. The prevalence of HT was significantly different among low, normal, and high AMH groups (43.75%, 26.03%, and 29.79%, respectively; *p*=0.035). The difference in the TgAb-positive rate between women with low AMH (35.42%), normal AMH (19.59%), and high AMH (23.40%) was statistically significant (*p*=0.040). However, the prevalence was comparable for positive TPOAb in low (31.25%), normal (20.36%), and high (23.40%) AMH groups, *p*=0.216 (Table 2). What stands out is TgAb levels and TgAb-positive rates were significantly different between different AMH level groups; the highest TgAb level and the TgAb-positive rate were shown in the low AMH group.

In multivariate logistic regression, TgAb was significantly associated with low AMH, so that with one unit increase in TgAb levels, the odds of having AMH below the 10th percentile of the values increase by 1.012 times (*P*=0.032) (Table 4). However, TSH, TPOAb, and age were not associated with low AMH (*P* > 0.05) (Table 4).

## 4. Discussion

Whether thyroid autoimmunity affects ovarian reserve, and if so, how it is affected, is currently unknown. This study provides us with an opportunity to examine the associations between AMH and HT in reproductive aged women. This study confirmed that the presence of antithyroid antibodies may play an important role in female infertility related to thyroid autoimmunity. We found that women with lower AMH levels had higher TgAb levels. In other words, TgAb levels are negatively correlated with AMH.

The cause of POF is unclear, but it may involve genetics, autoimmune, vaccination, enzymatic, oncologic treatment, environmental, and unknown factors [26]. Some studies report that autoimmunity is responsible for approximately 4–30% of POF cases [27, 28]. Previous studies have shown that there is an association between thyroid autoimmunity and infertility [29, 30]. Human and animal studies have shown that there is a negative correlation between ovarian reserve and thyroid autoimmunity [31, 32]. A case-control study found that AITD is independently associated with AMH and that women with AITD have lower AMH than in the control group [33]. In contrast, in another large retrospective cross-sectional analysis, it was reported that thyroid autoimmunity is not associated with low ovarian reserve [34].

In our study, of the 48 patients with low AMH levels, 17 were positive for TgAb antibodies (35.42%) and 15 were positive for TPOAb antibodies (31.25%). Further comparison revealed that there were no significant differences in the levels of TPOAb between the low, normal, and high AMH groups. Most studies have investigated the prevalence of autoimmune thyroid disease in infertile women and found that AITD is only related to the presence of TPOAb and not to the presence of TgAb [4, 6]. However, in this study, we found that women with low AMH levels had higher TgAb levels and a higher rate of TgAb positivity. AMH was inversely correlated with TgAb levels. The presence of TgAb may have a negative impact on the ovarian reserve. A study reported that serum AMH in HT patients is positively correlated with TPOAb and TgAb levels, while serum AMH had no correlation with TSH levels [21]. Kuroda et al. [20] demonstrated that the AMH level in patients with Hashimoto's disease increased significantly after levothyroxine supplementation, but a similar effect was not observed in cases of subclinical hypothyroidism. The AMH level in patients who were TgAb-positive and TPOAb-negative also significantly increased after levothyroxine treatment. Therefore, TgAb may have a key adverse impact on ovarian function. According to the Endocrine Society guidelines, infertility is one of the risk factors that should be considered when selecting targeted screening. It is recommended to add TgAb presence detection to increase the evidence of supporting autoimmunity screening [35]. We consider that, probably due to the extra cost of TgAb testing, most of the studies on the relationship between thyroid autoimmunity and ovarian reserve did not include TgAb testing or did not consider TgAb as a possible risk factor.

The underlying mechanism linking thyroid autoimmunity and ovarian reserve is not yet clear. In organ-specific and systemic autoimmune diseases, the ovary is a common target for autoimmune attacks [26, 36]. Approximately 10%–55% of patients with POF have related autoimmune diseases [36]. One study reported that TgAb and TPOAb can be detected in follicular fluid samples of women with thyroid autoimmunity, while antithyroid antibodies were not detected in follicular fluid specimens of women without thyroid autoimmunity. More importantly, follicular fluid TgAb levels were positively correlated with serum TgAb levels [37]. Premature ovarian insufficiency is an autoimmune disease, the numbers of effector Treg cells decreased, and CD4+ CD69+ activated T cells in peripheral blood increased [38]. The reduction of Treg cells is likely to lead to low tolerance of autoimmunity, which leads to the progression of POF. In addition to the decrease in Treg cells, there was an imbalance in the proportion of Th17/Treg cells in patients with POF [39].

Our study is one of the few to investigate the relationship between autoimmune thyroid disease, especially thyroid autoimmune antibodies (TPOAb and TgAb), and ovarian reserve, as quantified by AMH values. However, this study also has some limitations. First, other methods for evaluating ovarian reserve, such as FSH, estradiol, ovarian volume and follicle count, and inhibin B, were not tested in this study. AMH has been considered the most reliable biomarker. Second, the AMH value obtained in this study was determined by testing AMH once, and we did not assess ovarian reserve over time. Studies have shown that AMH levels remain almost unchanged from cycle to cycle and are stable. Third, age stratification was not performed; future studies should pay more attention to the age stratification and should be conducted longitudinally, covering the period of adolescence and adulthood. Fourth, sample size is not big enough yet, as our paper is only a preliminary study for AMH and autoimmune thyroid diseases, and we will conduct more work in our further research. Fifth, the ethnicity of the women in our research were not considered.

## 5. Conclusion

HT may occur more frequently in patients with a low AMH level, or patients with HT are more likely to have low AMH levels. However, the underlying mechanism and causality are not clear. In this study, it was concluded that low AMH is associated with high TgAb levels, and it is assumed that ovarian function may be affected by high TgAb levels. If the hypothesis presented in this study is true, it may provide therapeutic strategies for improving ovarian function. Further research to quantify the relative contribution of TgAb to AMH levels may provide a more comprehensive understanding of this basic biological process.

## Figures and Tables

**Table 1:** Baseline characteristics of study subjects.

	Total (*n* = 483)	Low AMH group (*n* = 48)	Normal AMH group (*n* = 388)	High AMH group (*n* = 47)	*p* values
Age (years)	33.77 ± 7.47	34.88 ± 5.92	33.61 ± 7.32	32.14 ± 4.89	0.819
TSH (*μ*IU/mL)	2.68 (1.78–4.24)	3.02 (1.46–4.32)	2.69 (1.81–4.21)	2.31 (1.70–4.18)	0.619
FT4 (ng/dl)	1.26 ± 0.45	1.22 ± 0.29	1.25 ± 0.44	1.33 ± 0.63	0.482
FT3 (pg/ml)	3.15 ± 1.56	3.11 ± 0.56	3.12 ± 1.59	3.38 ± 1.99	0.568
TPOAb (IU/mL)	13.48 (9.02–26.38)	14.96 (8.66–119.93)	13.33 (8.87–24.93)	15.06 (10.24–25.72)	0.394
TgAb (IU/mL)	13.56 (10.0–82.06)	37.62 (13.10–232.68)^*∗*^^#^	12.46 (10.0–67.04)	13.61 (10.0–95.74)	≤0.01
AMH (ng/mL)	1.72 (0.33–4.27)	0.01 (0.01–0.024)	1.73 (0.53–3.83)	9.08 (7.91–11.71)	≤0.01

TSH, thyroid-stimulating hormone; FT4, free thyroxine; FT3, free triiodothyronine; TPOAb, thyroid peroxidase antibody; TgAb, thyroglobulin antibody; AMH, anti-Müllerian hormone. *Note.* Rank the TgAb data and use the rank order as the measurement variable for ANOVA analysis, there is a statistical difference, and *p* value is 0.001. Furthermore, pairwise comparison results show that ^*∗*^a statistical difference between low and normal ovarian reserve (*p* < 0.001); ^#^a statistical difference between low and high ovarian reserve (*p*=0.039).

**Table 2:** Prevalence of thyroid dysfunction and thyroid autoimmune disease according to the level of AMH.

	Low AMH group (*n* = 48)	Normal AMH group (*n* = 388)	High AMH group (*n* = 47)	*p* values
Overt hypothyroidism, *n*(%)	2 (4.17%)	9 (2.32%)	1 (2.13%)	0.764
Subclinical hypothyroidism, *n*(%)	15 (31.25%)	97 (25%)	11 (23.4%)	0.608
Hashimoto's thyroiditis, *n*(%)	21 (43.75%)	101 (26.03%)	14 (29.79%)	0.035
TPOAb-positive women, *n*(%)	15 (31.25%)	79 (20.36%)	11 (23.4%)	0.216
TgAb-positive women, *n*(%)	17 (35.42%)	76 (19.59%)	11 (23.4%)	0.040

AMH, anti-Müllerian hormone; TPOAb, thyroid peroxidase antibody; TgAb, thyroglobulin antibody.

**Table 3:** Correlation analysis of AMH with thyroid function and thyroid autoantibodies.

	*γ*	*p* value
TSH	0.023	0.608
FT4	0.085	0.060
FT3	0.072	0.113
TPOAb	−0.024	0.599
TgAb	−0.114	0.013

TSH, thyroid-stimulating hormone; FT4, free thyroxine; FT3, free triiodothyronine; TPOAb, thyroid peroxidase antibody; TgAb, thyroglobulin antibody; AMH, anti-Müllerian hormone.

**Table 4:** Relative impact of demographic and clinical variables on AMH on the regression model.

Factors affecting AMH	Odd ratios	95% confidence interval		*p* value
		Lower limit	Upper limit	
Age	0.765	0.526	1.058	0.152
TSH	1.016	0.846	1.112	0.316
TPOAb	0.981	0.898	1.009	0.246
TgAb	1.012	1.002	1.041	0.032

TSH, thyroid-stimulating hormone; TPOAb, thyroid peroxidase antibody; TgAb, thyroglobulin antibody; AMH, anti-Müllerian hormone.

## Data Availability

The data used to support the findings of this study are available from the corresponding author upon request.
